# Authenticity analyses of Rhizoma Paridis using barcoding coupled with high resolution melting (Bar-HRM) analysis to control its quality for medicinal plant product

**DOI:** 10.1186/s13020-018-0162-4

**Published:** 2018-02-08

**Authors:** Bao-Zhong Duan, Ya-Ping Wang, Hai-Lan Fang, Chao Xiong, Xi-Wen Li, Ping Wang, Shi-Lin Chen

**Affiliations:** 1 College of Pharmaceutical Science, Hubei University of Chinese Medicine, Wuhan, 430065 China; 2 Institute of Chinese Materia Medica, China Academy of Chinese Medical Sciences, Beijing, 100700 China; 3 College of Pharmaceutical Science, Dali University, Dali, 671000 China; 4 Centre for Cancer and Inflammation Research, School of Chinese Medicine, Hong Kong Baptist University, Hong Kong, China

**Keywords:** Authentication, Rhizoma Paridis, DNA barcoding, High resolution melting, Adulterations, Natural health products

## Abstract

**Background:**

Rhizoma Paridis (Chonglou) is a commonly used and precious traditional Chinese medicine. *Paris polyphylla* Smith var. *yunnanensis* (Franch.) Hand. -Mazz. and *Paris polyphylla* Smith var*. chinensis* (Franch.) Hara are the two main sources of Chonglou under the monograph of Rhizoma Paridis in Chinese Pharmacopoeia. In the local marketplace, however, this medicine is prone to be accidentally contaminated, deliberately substituted or admixed with other species that are similar to Rhizoma Paridis in shape and color. Consequently, these adulterations might compromise quality control and result in considerable health concerns for consumers. This study aims to develop a rapid and sensitive method for accurate identification of Rhizoma Paridis and its common adulterants.

**Methods:**

DNA barcoding coupled with high resolution melting analysis was applied in this research to distinguish Rhizoma Paridis from its adulteration. The internal transcribed spacer 2 (ITS2) barcode was selected for HRM analysis to produce standard melting profile of the selected species. DNA of the tested herbal medicines was isolated and their melting profiles were generated and compared with the standard melting profile of *P. polyphylla* var. *chinensis*.

**Results:**

The results indicate that the ITS2 molecular regions coupled with HRM analysis can effectively differentiate nine herbal species, including two authentic origins of Chonglou and their seven common adulterants. Ten herbal medicines labeled “Chonglou” obtained from a local market were collected and identified with our methods, and their sequence information was analyzed to validate the accuracy of HRM analysis.

**Conclusions:**

DNA barcoding coupled with HRM analysis is a accurate, reliable, rapid, cost-effective and robust tool, which could contribute to the quality control of Rhizoma Paridis in the supply chain of the natural health product industry (NHP).

**Electronic supplementary material:**

The online version of this article (10.1186/s13020-018-0162-4) contains supplementary material, which is available to authorized users.

## Background

Rhizoma Paridis (Chonglou) is a precious traditional Chinese medicine. It originates from the dried rhizomes of both *Paris polyphylla* Smith var. *yunnanensis* (Franch.) Hand. -Mazz. and *P. polyphylla* Smith var. *chinensis* (Franch.) Hara, according to the Chinese Pharmacopoeia [1]. This medicine is most notably an ingredient of the hemostasis prescription ‘Yunnan Baiyao’ [2]. It plays an important clinical role in treating snake bites, fractures, parotitis, tumors, analgesia and traumatic bleeding. Recent pharmacological studies have indicated that Chonglou exhibits a variety of pharmacological activities, including anti-cancer, anti-inflammatory, antibacterial and immuno-regulatory activities [3]. Unfortunately, slow growth and over-harvesting for the past several years have led to a significant decline in Chonglou’s population and caused it to be listed as a vulnerable species by the IUCN (International Union for Conservation of Nature and Natural Resources). The lack of supply and subsequent high price can result in adulteration of Chonglou, including accidentally contamination, deliberately substitution or admixture with other species [4]. More specifically, Chonglou is often contaminated with several common adulterants: *P. thibetica* Franch, *P. tengchongensis* Y.H. Ji, C.J. Yang & Y.L. Huang, *P. forrestii* (Takht.) H. Li, *P. mairei* H. Lév, *Tupistra* spp., *Trillium tschonoskii* Maxim. and *Polygonum paleaceum* Wall. These adulterants are usually of poor quality and some might even be toxic. Contaminated with these plants can pose considerable health threat for the consumers [5]. It was reported that several *Tupistra* species can induce headache and vomiting [6]. Thus, Chonglou authenticity is not only an issue of medicinal quality but also of safety. A fast, reliable and robust method is urgently needed to be established for the herbal industry and regulatory agencies to identify Chonglou species from their adulterants.

With rapid advances in sequencing technologies, Herbgenomics provides an effective platform to support biological analyses of complex herbal products [7, 8]. It is now being widely applied to DNA-based herbal identification through the establishment of an herbal gene bank. DNA-based methods have gained popularity for unequivocal plant species identification because DNA molecules can be found in all tissues and they cannot be affected by environment or physiological factors [9–13]. DNA barcoding, a method that use the sequence of a standard region of DNA for the purpose of species identification, has been recognized as a powerful tool for the identification of medicinal plants and their adulterants at the species level [4, 14–17]. However, DNA sequencing of each individual sample is expensive, time consuming and cannot easily be applied to studies of large samples because of financial constraints [18]. High resolution melting (HRM) analysis is a closed-tube post-PCR analysis that can differentiate between samples even with a single base change in DNA sequences. This technique permits the genotyping and the quantification of adulterants and has been proved to be fast, reliable, low-cost in terms of consumables [19, 20]. Very recently, the Bar-HRM analysis has been introduced for species identification and authentication. The use of just Bar-HRM for species authentication and detection of adulteration in commercial products has been reported [21, 22]. To date, it has not been applied as a tool for the species authentication of Chonglou.

The goal of this research is to develop a rapid, scientifically rigorous and accurate method to identify and authenticate two types of Chonglou, namely *P. polyphylla* var. *yunnanensis* and *P. polyphylla* var. *chinensis*, from their adulterants. For that purpose, Bar-HRM analysis using universal DNA barcoding ITS2 region was conducted. The proposed methodology was further applied to differentiate ten herbal medicines labeled as Chonglou bought in market place. This will provide the first evidence for the utility of Bar-HRM analysis to test the authenticity of Chonglou sold in the local market place.

## Methods

### Reference plant material and DNA extraction

Thirty two fresh rhizome samples from nine species were collected from the Yunnan Province, People’s Republic of China, including *P. polyphylla* var. *yunnanensis*, *P. polyphylla* var. *chinensis*, *P. thibetica*, *P. tengchongensis*, *P. forrestii*, *P. mairei*, *Tupistra* spp., *T. tschonoskii* and *P. paleaceum* (Table 1). The rhizomes were gently washed and dried at 50 °C. All of the materials were authenticated by Professor Conglong Xia of the College of Pharmaceutical Science, Dali University. In addition, ten herbal medicines labeled “Chonglou” were purchased from a local market in the Yunnan Province. All voucher specimens and herbal medicines were deposited in the Herbarium of Dali University (DLU). Samples taken from dried rhizomes (30 mg) were rubbed for 2 min at a frequency of 30 r/s. Extraction proceeded by using the modified Plant Genomic DNA Extraction Kit (Tiangen Biotech Co., Ltd., Beijing, China). The DNA was diluted to 50 ng/μL and later stored at − 20 °C for further use.

**Table 1:** The origin of the plant species used in the study and their GenBank accession numbers

No.	Family	Botanical name	Voucher number	Genbank accessions number
1	*Liliaceae*	*Paris polyphylla* var. *yunnanensis* (Franch.) Hand. -Mazz.	YN0001MT01, YN0001MT02, YN0001MT03, YN0001MT04, YN0001MT05, YN0001MT06	KX458130, KX458131, KX458132, KX458133, KX458134, KX458135
2		*Paris polyphylla* var. *chinensis* (Franch.) H. Hara	YN0011MT01, YN0011MT02, YN0011MT03	KX458140, KX458141, KX458142
3		*Paris thibetica* Franch.	YN0012MT01, YN0012MT02, YN0012MT03	KX458136, KX458137, KX458138, KX458139
4		*Paris tengchongensis* Y.H. Ji, C.J. Yang & Y.L. Huang	YN0007MT01, YN0007MT02, YN0007MT03, YN0007MT04, YN0007MT05, YN0007MT06	KX458121, KX458122, KX458123, KX458124, KX458125, KX458126
5		*Paris forrestii* (Takht.) H. Li	YN0009MT01, YN0009MT02, YN0009MT03	KX458127, KX458128, KX458129
6		*Paris mairei* H. Lév	YN0005MT01, YN0005MT02, YN0005MT03	KX458118, KX458119, KX458120
7		*Tupistra* spp. (not define)	YN0016MT03, YN0016MT04, YN0016MT05	/
8		*Trillium tschonoskii* Maxim.	YN0016MT10, YN0016MT11, YN0016MT12	KX458143, KX458144, KX458145
9	*Polygonaceae*	*Polygonum paleaceum* Wall.	YN0016MT01, YN0016MT02	KX458146, KX458147

### HRM-PCR amplification

A conserved region of the ITS2 region of nuclear ribosomal DNA was used as the optimal region for the assay because of it has previously correctly identified the medicinal plants of the genus *Paris* [23]. DNA was amplified using the polymerase chain reaction (PCR). The real-time PCR mixture (25 μL) contained 50 ng of genomic DNA, 12.5 μL of 2 × HRM PCR master mix, 1 μL forward (5′-ATGCGATACTTGGTGTGAAT-3′) primer (10 μmol/L), and 1 μL reverse (5′-GACGCTTCTCCAGACTACAAT-3′) primer (10 μmol/L), and distilled water up to the final volume. Real-time PCR was performed in a Rotor-Gene Q MDx cycler (Qiagen, Hilden, Germany) under the following conditions: 94 °C for 3 min followed by 40 cycles of 94 °C for 30 s, 56 °C for 30 s and 72 °C for 45 s. The fluorescent data were acquired at the end of each extension step of the PCR cycle. HRM was performed as follows: pre-melt at 95 °C for 1.5 min, followed by a temperature raise from 65 to 95 °C with 0.1 °C degree increments and a 2 s hold time for each acquisition step.

The Rotor-Gene Q software (Qiagen, Hilden, Germany) was used to genotype the different varieties and the herbal products. The negative derivative of the fluorescence (*F*) over temperature (*T*) (*dF*/*dT*) curve displayed the melting temperature (*T*m), and the normalized raw curve depicted the decreasing fluorescence against increasing temperature. Pre- and post-melt normalization regions were set to define the temperature boundaries of the normalized and difference plots and to generate normalized melting curves of the normalized and difference plots [24]. *P. polyphylla* var. *chinensis* was also used as reference species. To assess the discriminative power of Bar-HRM analysis, samples from both the fresh plants and dry tissue were used for PCR amplification and HRM analysis.

### Sequencing and data analysis

To verify the HRM analysis results, all the PCR products were directly sequenced in two directions using the automated ABI 3730 XL sequencer (Applied Biosystems, Foster City, CA, USA). Sequence results were submitted to the GenBank database (accession numbers were listed in Table 1), assembled with CodonCode Aligner 5.1.5 (CodonCode Co., USA) and aligned using Clustal W. The neighbor-joining (NJ) trees were calculated and constructed using the MEGA 5.05 software with the Bootstrap method (1000 resembling) and the K2P model [25].

The Minimum Standards of Reporting Checklist contains details of the experimental design, and statistics, and resources used in this study (Additional file 1).

## Results

### Barcoding of PCR products by HRM analysis

**Fig. 1:**
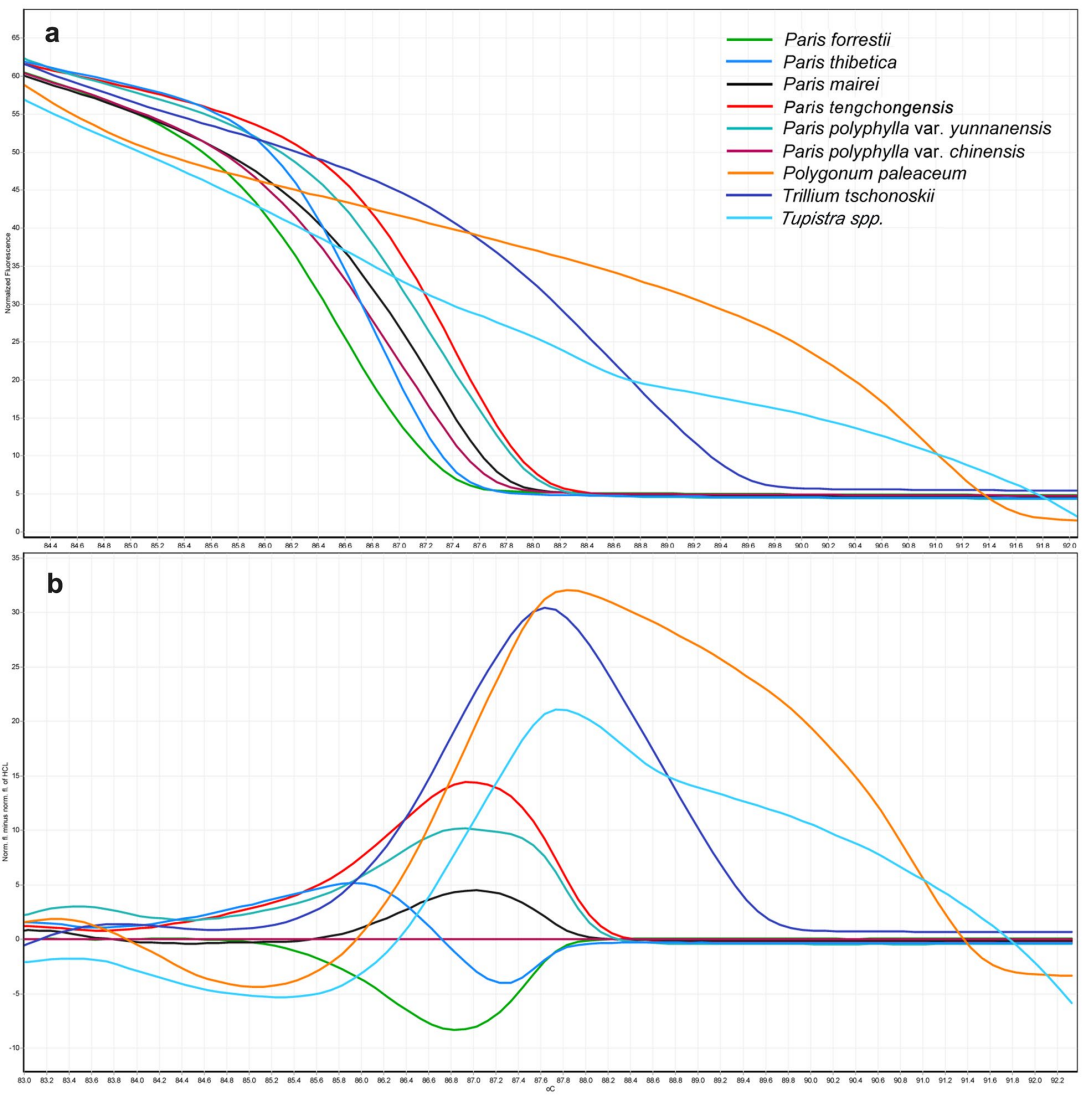
**a** The normalized melting profiles of nine plant species obtained by HRM analysis with ITS2 as the universal marker. **b** The difference graphs of nine species using *Paris polyphylla* var. *chinensis* as a reference genotype. A color-code table listing the species was used

The amplicons of ITS2 were analyzed with HRM to define their *T*ms. There exist some differences in ITS2 region among Chonglou and their adulterate species reflected by their *Tm* values (Table 2). Although the *T*ms value of *Paris* genus were close to each other, each species still could be clearly discerned by the plots of the normalized melting curves (Fig. 1). Average genotype confidence percentages (GCPs) were also calculated and a cut-off value of 90% was used to assign a genotype for each region. The GCPs from the HRM analyses of the ITS2 region of nine species are listed in Table 3. As shown in Table 3, all species were sufficiently and confidently identified with a value of 100%. The highest GCP (81.28%) was found between the *P. mairei* and *P. polyphylla* var. *chinensis* species, while the lowest (0.0) was between *Paris* and the other genera. The melting profiles of the ITS2 amplicons from the nine species were assessed by plotting two different curves: the derivate melt curve (Fig. 1a) and the difference plot melt curve (Fig. 1b). The normalized HRM curves with the barcode marker ITS2 showed that all the nine species could be easily and visually distinguished.

**Table 2:** The values of the melting temperatures of nine species measured by HRM analysis using ITS2 as marker

No.	Botanical name	Examin	Tm (°C)
1	*Paris polyphylla* var. *yunnanensis* (Franch.) Hand. -Mazz.	6	87.31 ± 0.07
2	*Paris polyphylla* var. *chinensis* (Franch.) H. Hara	3	86.88 ± 0.02
3	*Paris thibetica* Franch.	3	86.84 ± 0.02
4	*Paris tengchongensis* Y.H. Ji, C.J. Yang & Y.L. Huang	6	87.55 ± 0.04
5	*Paris forrestii* (Takht.) H. Li	3	86.68 ± 0.04
6	*Paris mairei* H. Lév	3	87.37 ± 0.03
7	*Tupistra* spp. (not define)	3	92.66 ± 0.08
8	*Trillium tschonoskii* Maxim.	3	88.91 ± 0.17
9	*Polygonum paleaceum* Wall.	2	91.02 ± 0.02

**Table 3:** The results of average genotype confidence percentages from the HRM analysis of nine species

Taxon	*P. forrestii*	*P. tengchongensis*	*P. mairei*	*P. polyphylla* var. *chinensis*	*P. polyphylla* var. *yunnanensis*	*P. thibetica*	*Trillium tschonoskii*	*Polygonum paleaceum*	*Tupistra* spp.
*P. forrestii*	100								
*P. tengchongensis*	0.13	100							
*P. mairei*	19.11	16.85	100						
*P. polyphylla* var. *chinensis*	48.71	4.55	*81.28*	100					
*P. polyphylla* var. *yunnanensis*	1.24	72.1	44.33	18.75	100				
*P. thibetica*	28.57	3.32	47.6	63.51	16.66	100			
*Trillium tschonoskii*	0	0.06	0	0	0.01	0	100		
*Polygonum paleaceum*	0	0	0	0	0	0	0.04	100	
*Tupistra* spp.	0	0.02	0.02	0.01	0.02	0	0.94	0.6	100

The highest GCP was found between the P. mairei and P. polyphylla var. chinensis, which are presented in italics

### Species authentication using DNA sequencing

**Fig. 2:**
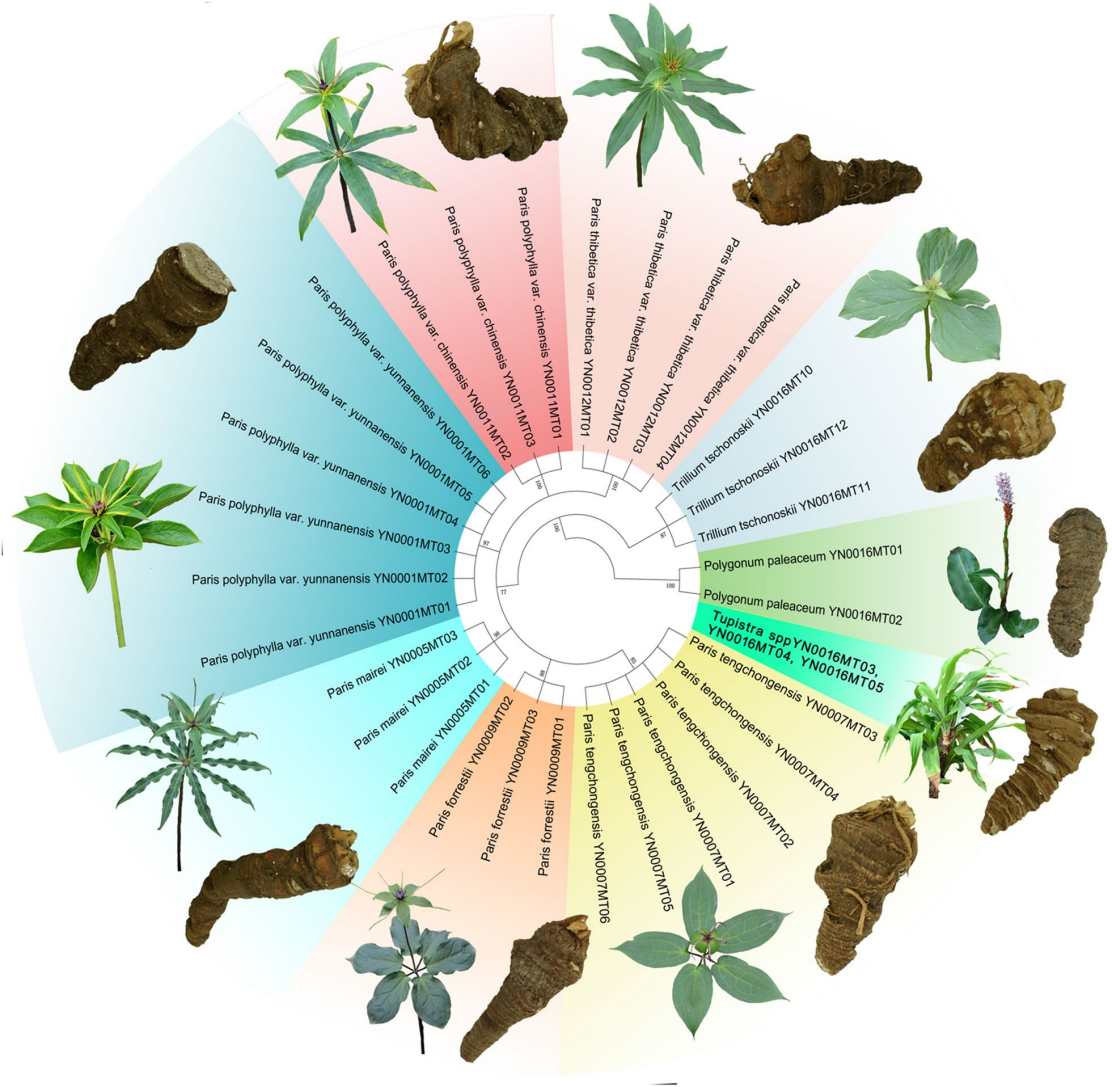
The phylogenetic tree of Rhizoma Paridis and its adulterants constructed from the ITS2 sequences using the N-J method (Bootstrap scores ≥ 50%). Representatives of the species illustrate the morphological variation in *Paris* and related species

**Fig. 3:**
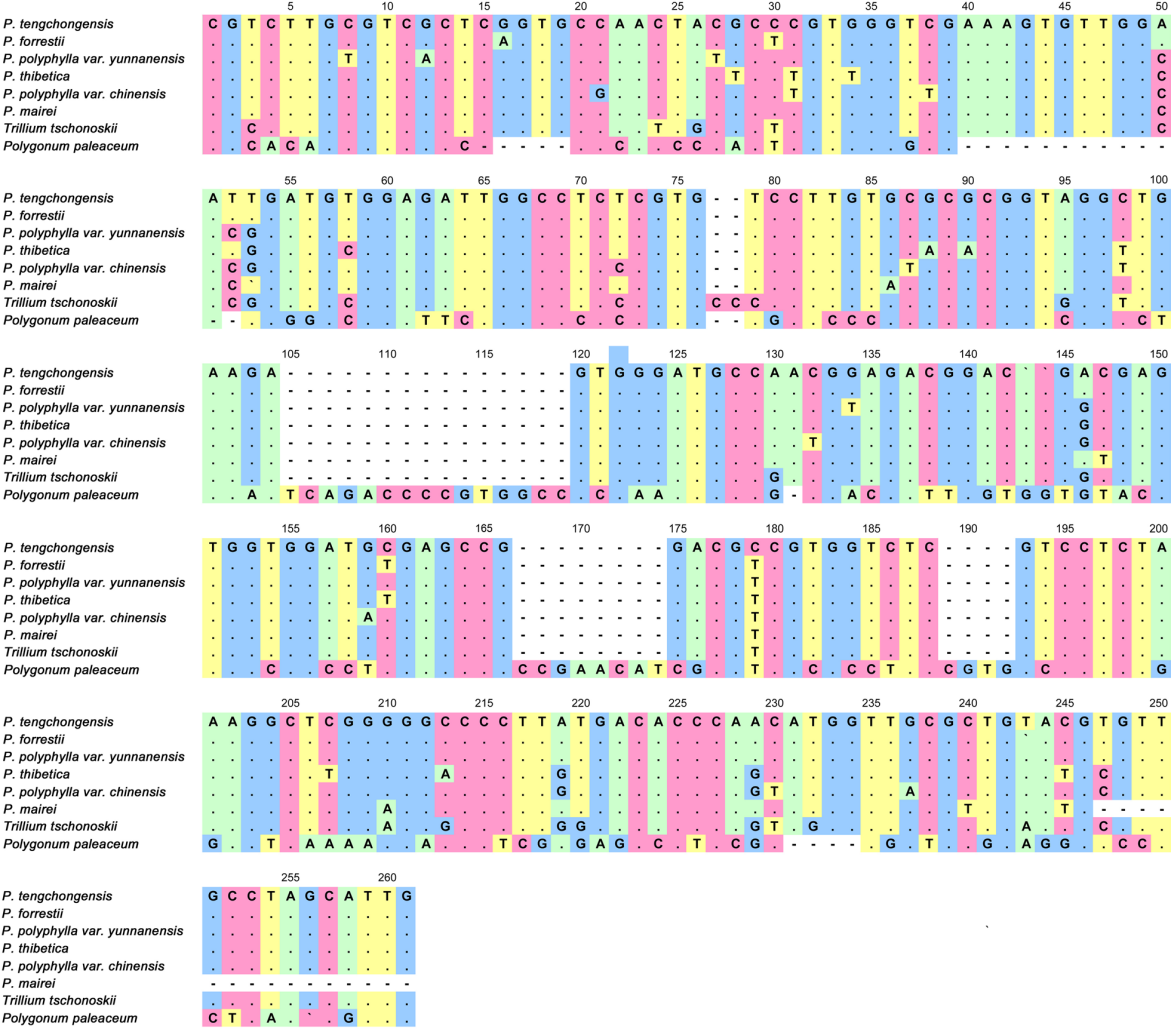
The DNA sequence alignment analysis of nine plant species showing differences in the DNA level alignment confirmed the Bar-HRM results

To validate the results of HRM analysis, amplicon sequences of all samples from the nine reference plant species were sequenced. Twenty-seven ITS2 sequences from eight species were successfully obtained, except for *Tupistra* spp. These sequences were used to construct phylogenetic tree [neighbor-joining (NJ)]. Eight species can be clearly distinguished by the NJ tree (Fig. 2), namely *P. polyphylla* (var. *yunnanensis* and var. *chinensis*), *P. thibetica*, *P. tengchongensis*, *P. forrestii*, *P. mairei*, *T. tschonoskii* and *P. paleaceum*. The genetic relationships reflected by the NJ tree were in good agreement with the Bar-HRM results. The sequence of each amplicon and the alignment among eight species are shown in Fig. 3. Sequencing results revealed that the differences in the melting curves maybe caused by deletions, insertions and a number of SNPs in the ITS2 region. In brief, the variations of the eight species in ITS2 region are capable to produce differences in the HRM analysis profile.

### Application of Bar-HRM to commercial herbal medicine

**Fig. 4:**
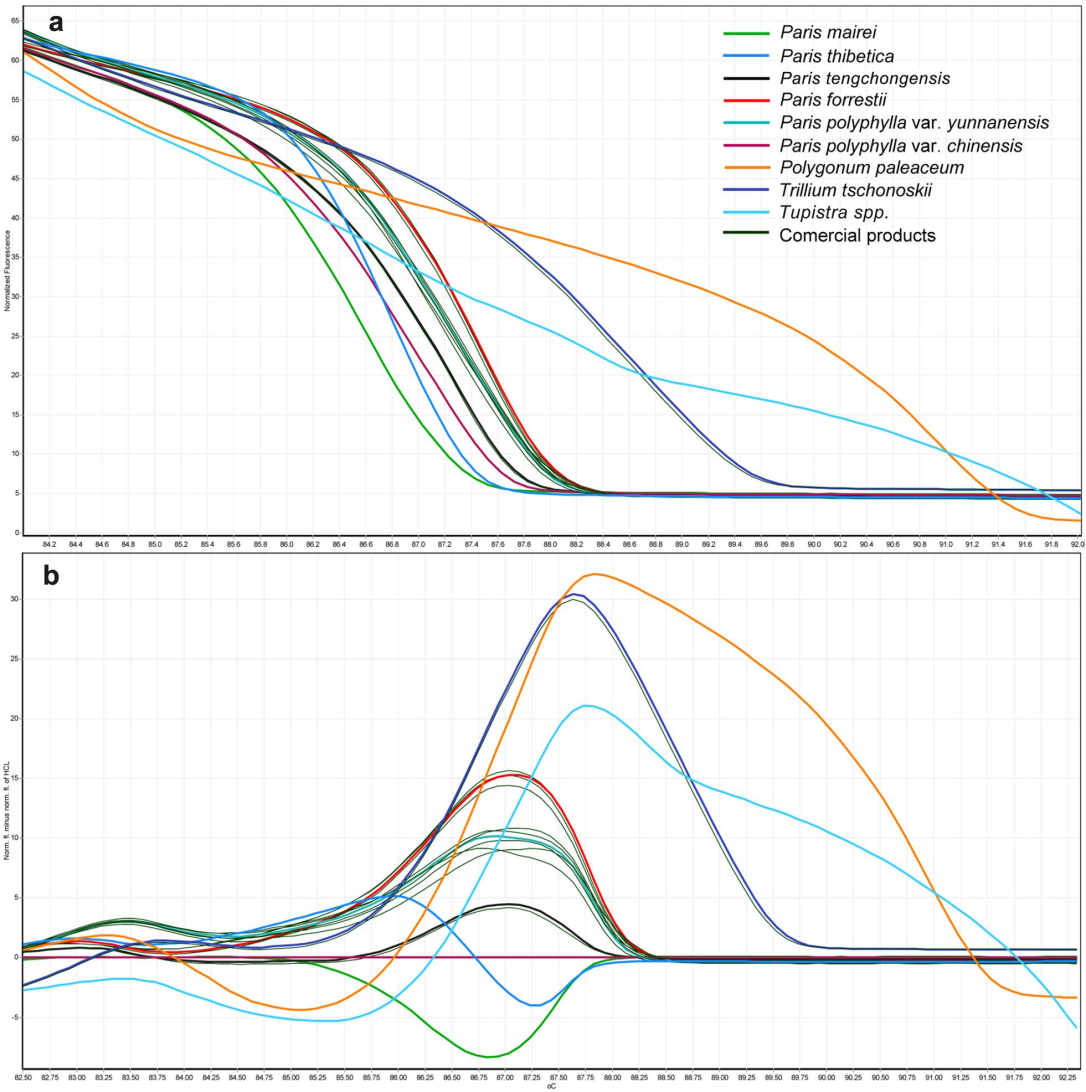
The Bar-HRM analysis of nine plant species and ten commercial Chonglou products using HRM analysis with the ITS2 marker. **a** Normalized melting curves. **b** Difference curves, with the mean *Paris polyphylla* var. *chinensis* curve used as a reference genotype. A color-code table listing the species was used

As mentioned previously, Chonglou sold in the local market places can be confused with certain other species that are similar in shape and color with the authentic Chonglou. To evaluate the accuracy of Bar-HRM method and to assure the quality of Chonglou in local market, the proposed methodology was further applied to authenticate ten batches of samples labelled as “Chonglou”. The HRM curves of nine reference plant species and ten samples are shown in Fig. 4. *T*m values and GCPs of ten samples obtained from the HRM analysis are shown in Tables 4 and 5. As shown in Fig. 4 and Tables 4 and 5, the *T*ms of five Chonglou samples (Com-1, Com-2, Com-3, Com-8, and Com-10) ranged from 87.29 to 87.33 °C, which agreed with the *T*m of *P. polyphylla* var. *yunnanensis*, and the curves overlapped completely. These results demonstrated that all these five samples were *P. polyphylla* var. *yunnanensis*. Among them, three samples (Com-4, Com-5, and Com-6) were identified as *P. tengchongensis.* Com-7 sharing a similar plot with *T. tschonoskii*. Com-9 was identified as *P. mairei.* These results indicate that Bar-HRM analysis can be effectively used to differentiate Chonglou from its adulterants.

**Table 4:** The list of ten commercial ‘‘Chonglou’’ products that were identified using ITS2 sequencing coupled with high resolution melting temperature values

Code	Asserted species	Tm (°C)	HRM
Com-1	Chong-Lou (not define)	87.30	*Paris polyphylla* var. *yunnanensis*
Com-2	Chong-Lou (not define)	87.29	*Paris polyphylla* var. *yunnanensis*
Com-3	Chong-Lou (not define)	87.32	*Paris polyphylla* var. *yunnanensis*
Com-4	Chong-Lou (not define)	87.57	*Paris tengchongensis*
Com-5	Chong-Lou (not define)	87.55	*Paris tengchongensis*
Com-6	Chong-Lou (not define)	87.52	*Paris tengchongensis*
Com-7	Chong-Lou (not define)	88.40	*Trillium tschonoskii* Maxim.
Com-8	Chong-Lou (not define)	87.31	*Paris polyphylla* var. *yunnanensis*
Com-9	Chong-Lou (not define)	87.39	*Paris mairei*
Com-10	Chong-Lou (not define)	87.33	*Paris polyphylla* var. *yunnanensis*

*Com* Commercial

**Table 5:** The results of genotype confidence percentages from the HRM analysis of ten commercial “Chonglou” products

Taxon	COM-1	COM-2	COM-3	COM-4	COM-5	COM-6	COM-7	COM-8	COM-9	COM-10
*P. forrestii*	0.95	2.38	1.62	0.19	0.1	0.22	0	1.05	21	1.84
*P. tengchongensis*	75.04	61.77	68.07	97.66*	99.39*	98.64*	0.09	76.7	14.91	61.04
*P. mairei*	39.09	57.09	49.88	19.82	14.73	21.98	0	41.96	99.73*	49.81
*P. polyphylla* var. *chinensis*	15.75	27.23	22.04	5.84	3.87	6.49	0	16.9	83.79	23.51
*P. polyphylla* var. *yunnanensis*	99.47*	95.99*	99.22*	79.11	69.88	78.62	0.02	99.21*	40.64	97.72*
*P. thibetica*	14.48	20.85	18.49	4.93	3.02	4.81	0	13.77	48.15	23.46
*Trillium tschonoskii*	0.01	0.01	0.01	0.04	0.06	0.04	*98.93*	0.02	0	0.01
*Polygonum paleaceum*	0	0	0	0	0	0	0.03	0	0	0
*Tupistra* spp.	0.02	0.03	0.02	0.02	0.02	0.02	1.12	0.02	0.02	0.01

*Over the 90% confidence percentage threshold are presented in italics

## Discussion

Species identification is critical to ensuring the quality of herbal medicine. HRM analysis is a method that measures the dissociation rates of double-stranded DNA into single-stranded DNA in increasing temperatures [26]. Minute difference in amplicon size or composition, even a simple nucleotide, can be detected. In general, different genotypes have their own unique transitions, including changes in curve shape and in the plots of their melting curves, which can be shown in their HRM profiles [24].

At present, several methods, such as high performance liquid chromatography (HPLC) and near infrared spectroscopy (NIR), have been introduced to discriminate the controversial *Paris* species [27, 28]. However, the HPLC method is relatively expensive and NIR spectroscopy suffers from low precision and tedious calibration, which is not ideal for the rapid screening of large numbers of samples. For these reasons, molecular biology-based analysis is becoming increasingly popular for the differentiation and identification of species in several scientific fields [29]. Among the various molecular biology techniques, DNA barcoding, ITS2, has been successfully applied to distinguish *Paris* species [23]. However, DNA sequencing technology is relatively expensive and time-consuming since the sequencing process is about 1–2 days. The use of Bar-HRM can overcome the cost and time issues (the whole process only takes 4 h). Moreover, this new approach has its own advantage compared with previously employed methods. The Bar-HRM analysis is performed in one completely “closed” tube and the results are available for analysis at the end of the run.

In this study, we have successfully applied Bar-HRM analysis for the rapid detection and identification of two major *Paris* species and their seven adulterants. The results indicated that Bar-HRM is a sensitive, fast, cheap, and reliable method for identifying and tracking herbal medicines. To validate the accuracy of polymorphisms detected by HRM analysis, the sequences of the reference plant specie were analyzed. Each PCR product was amplified and both strands were sequenced in opposite directions. Although the PCR products of *Tupistra* spp. were clearly visible as a single band on agarose gel, the sequence information of *Tupistra* spp. was not obtained, and there are no documented reports on its ITS2 sequences information in the GeneBank. The multi-copy of ITS2 region may lead to the failure of sequence. However, the HRM melting curves could be used to distinguish Chonglou from its adulterants clearly, included *Tupistra* spp. Considering these features, Bar-HRM can be a useful tool for quality control of herbal medicines in the supply chain of the natural health product industry (NHP) and medicine supervisory institutions. In conclusion, this study substantially contributes to the protection of public health worldwide.

## Conclusions

The Bar-HRM analysis has been used in this study to differentiate Chonglou, namely *P. polyphylla* var. *yunnanensis* and *P. polyphylla* var. *chinensis*, from their adulterate species. Species-specific PCR amplification of the ITS2 region was performed to validate the results of HRM analysis. The difference between Chonglou species and their adulterants can be observed from their sequencing information. The proposed method has been proved to be accurate, reliable, rapid, cost-effective and robust for identifying Chonglou from its adulterants. Bar-HRM analysis is also feasible for high-throughput assays, and could be well applied to other herbal species in the future, ensuring the perceived value and quality of herbal medicines.

## Additional file

**Additional file 1.** Minimum Standards of Reporting Checklist.

## Abbreviations

Bar-HRM: barcoding coupled with high resolution melting
HRM: high resolution melting
ITS2: internal transcribed spacer 2
IUCN: International Union for Conservation of Nature
GCPs: genotype confidence percentages
PCR: polymerase chain reaction
*T*m: melting temperature
HPLC: high performance liquid chromatography
NIR: near infrared spectroscopy
NHP: natural health product industry
